# Improvement of the Respiratory Function in a Patient With Mandibular Retrognathia and Hyperdivergent Skeletal Pattern With Condylar Resorption by Le Fort I Osteotomy and Genioplasty

**DOI:** 10.1155/crid/7229009

**Published:** 2025-12-22

**Authors:** Yukiho Kobayashi, Yunaho Yonemitsu, Namiaki Takahara, Tetsuya Yoda, Norihisa Higashihori, Keiji Moriyama

**Affiliations:** ^1^ Department of Maxillofacial Orthognathics, Division of Maxillofacial and Neck Reconstruction, Graduate School of Medical and Dental Sciences, Institute of Science Tokyo, Tokyo, Japan; ^2^ Department of Maxillofacial Surgery, Division of Maxillofacial and Neck Reconstruction, Graduate School of Medical and Dental Sciences, Institute of Science Tokyo, Tokyo, Japan; ^3^ Oral Science Center, Institute of Science Tokyo, Tokyo, Japan

**Keywords:** genioplasty, Le Fort osteotomy, mandibular condyles, obstructive sleep apnea, retrognathia

## Abstract

**Introduction:**

This case report describes the multidisciplinary treatment of a patient with mandibular retrognathia and hyperdivergent skeletal pattern, condylar resorption, and severe obstructive sleep apnea (OSA).

**Case Presentation:**

A 42‐year‐old female patient presented with a chief complaint of mandibular retrognathia. The patient had no significant medical history, and her body mass index was 18.8. She had a +9.0 mm overjet and +1.0 mm overbite, and an Angle Class II molar relationship. Cephalometric analysis revealed SNA, SNB, and FMA of 76.0°, 67.5°, and 50.5°, respectively. Computed tomography (CT) images showed severe bilateral mandibular condylar head deformity due to resorption. Polysomnography revealed an apnea–hypopnea index (AHI) of 43.3, leading to a diagnosis of severe OSA. Correspondingly, continuous positive airway pressure (CPAP) therapy was started immediately.

**Management and Outcomes:**

After 19 months of preoperative orthodontic treatment with preadjusted edgewise appliances, Le Fort I osteotomy was performed to impact the maxilla by 3.0 mm at the anterior and 4.0 mm at the posterior nasal spine. The mandible was autorotated 8.6° counterclockwise, reducing ANB from +8.5° to +2.0°. A genioplasty was also performed. After 8 months of postoperative orthodontic treatment, the AHI decreased to 2.3, and CPAP therapy was discontinued due to significant improvement in respiratory function. CT images showed an increase in the upper airway volume after orthognathic surgery. No remarkable morphological changes were observed in the mandibular condylar head during orthodontic treatment. Favorable occlusion was maintained with no apparent relapse after 21 months of retention.

**Discussion:**

Unlike conventional maxillomandibular advancement for OSA, the surgical method described in this case study combines counterclockwise mandibular rotation and genioplasty to simultaneously improve respiratory function and craniofacial morphology in patients with hyperdivergent and Class II skeletal pattern accompanied by mandibular condylar head deformity.

**Summary**

• A retrognathic case with obstructive sleep apnea (OSA) was managed using a multidisciplinary approach.
• Le Fort I osteotomy achieved counterclockwise mandibular rotation.
• A favorable occlusion and facial profiles were established.
• Increased airway volume and improved respiratory function were achieved.
• No evidence of condylar resorption progression was observed during treatment.
• This approach differs from conventional surgery for patients with OSA.

## 1. Introduction

Sleep apnea (SA) is a common sleep‐related breathing disorder that occurs in all age groups. There are two types of SA: OSA, in which the patient attempts to breathe during apnea and usually snores, and central SA, in which no attempt is made to breathe [1]. The apnea–hypopnea index (AHI) is the total number of apnea and hypopnea events per hour of sleep. An AHI of ≥ 5 but < 15 is considered mild, ≥ 15 but < 30 is considered moderate, and ≥ 30 is considered severe [1, 2]. Obesity, aging, being male, and abnormalities in the skeletal morphology of the craniofacial region are associated with OSA onset [1]. Sleep‐related breathing disorder are reportedly involved in the development and exacerbation of various cardiovascular diseases [3].

Treatment options for OSA include continuous positive airway pressure (CPAP) and oral appliance therapies, weight loss, nasal or pharyngeal airway surgery, and maxillofacial and orthognathic surgery. CPAP is typically recommended as the primary treatment for moderate‐to‐severe OSA [1]. A recently developed medical device stimulates the hypoglossal nerve—responsible for controlling the genioglossus muscle—exclusively during inhalation while asleep [4]. Its effectiveness has been validated, and it is now being introduced in clinical practice. Furthermore, diminished lung capacity during supine sleep may contribute to increased upper airway collapsibility during sleep. As a female‐specific factor, menopause‐related declines in estrogen and progesterone have also been shown to exacerbate OSA [5].

Craniofacial morphology significantly influences OSA development in Asian populations [6, 7]. Generally, patients with OSA possess a smaller anatomical upper airway diameter than healthy individuals [8–10] along with markedly reduced pharyngeal airway volume [8], inferior positioning of the hyoid bone, and increased anterior facial height due to clockwise mandibular rotation [11, 12]. The relationship between occlusion and respiratory function correlates with an anterior overjet of ≥ +6 mm and OSA development [13].

The pathogenesis of OSA has been attributed to factors including soft tissue deposits around the upper airway, craniofacial anatomy, tongue size, and tonsillar hypertrophy. Among these, obesity‐related soft tissue accumulation around the pharynx is considered a major factor affecting upper airway diameter. Dong et al. reported that a narrower upper airway width is associated with a higher AHI, while a more inferiorly positioned hyoid bone relative to the mandible negatively correlates with the lowest nocturnal oxygen saturation [14].

Nevertheless, as OSA manifests exclusively during sleep, anatomical narrowing alone cannot fully account for its pathophysiological. The pharynx′s flexibility enables efficient swallowing and clear speech. To maintain airway patency against inspiratory negative pressure, the genioglossus muscle exhibits increased activity during inhalation and diminished activity during exhalation [15]. Dysfunction of this mechanism, combined with anatomical narrowing of the upper airway, leads to OSA development [16]. Recent advances include a medical device designed to stimulate the hypoglossal nerve—which innervates the genioglossus muscle—during sleep‐associated inhalation [4]. Its efficacy has been demonstrated, and clinical applications are underway. Additionally, decreased lung capacity in the supine position may increase upper airway collapsibility during sleep. As a female‐specific factor, reduced levels of estrogen and progesterone following menopause have been identified as factors exacerbating OSA [5].

The direct relationship between craniofacial morphology and function has long been a focus in orthodontic research. Currently, there are no clear guidelines regarding the indications for when maxillomandibular advancement (MMA) or orthognathic surgery should be performed in skeletal Class II cases with retruded mandibles. Holty and Guilleminault showed that the greater the maxillary advancement, the greater the improvement in AHI [17]. However, considerable forward movement of the maxilla and mandible in Asian patients may negatively impact facial aesthetics. Additionally, SA‐related incidents have become a social issue [18], whereas the diagnosis, treatment, management, and prevention have become important issues for medical professionals. Clarifying the relationship between craniofacial morphology and respiratory function will lead to improvements in orthodontic diagnosis.

This case report describes the multidisciplinary retreatment of a patient who presented with mandibular retrognathia, hyperdivergent skeletal pattern, severe OSA, and condylar resorption. The approach included managing respiratory function, pre‐ and postoperative orthodontics, orthognathic surgery (Le Fort I osteotomy and genioplasty), and ongoing monitoring of the temporomandibular joint (TMJ).

## 2. Case Presentation

A 42‐year‐old woman was referred to the Orthodontic Department, Institute of Science Tokyo Hospital, with a chief complaint of mandibular retrognathia. Her body mass index was 18.8, within the normal range. The patient had no remarkable medical history. She had a symmetrical frontal view with excessive mentalis muscle tension and lip‐closure disorder (Figure 1) and presented with a retrognathic profile (Figure 1). An intraoral examination revealed an Angle Class II molar relationship with moderate crowding of the anterior mandibular incisors (Figure 1). The overjet and overbite were +9.0 and +1.0 mm, respectively (Figure 1). The maxillary midline coincided with the facial midline, and the mandibular midline deviated 1.0 mm to the right (Figure 1). She had undergone orthodontic treatment to correct maxillary protrusion in her teenage years at another university hospital, where her bilateral maxillary and mandibular first premolars were extracted. Her right mandibular second molar was also extracted due to caries, and an extension bridge was placed using her right mandibular second premolar and right first molar as abutments (Figure 1). Orthopantomograms showed several teeth undergoing root canal treatment (Figure 1d). Periapical radiolucency was also observed, and several crown restorations had been placed (Figure 1). Cephalometric analysis revealed a clockwise mandibular rotation (FMA, 50.5°), resulting in a skeletal Class II malocclusion (SNA, 76.0°; SNB, 67.5°; and ANB, +8.5°; Table 1). The maxillary and mandibular incisors were lingually inclined (U1 to FH plane, 107.0°; IMPA, 87.1°; Table 1).

Figure 1Pretreatment (T1) records. (a) Facial photographs. (b) Intraoral photographs. (c) Lateral and anteroposterior cephalograms. (d) Orthopantomograms. (e) 3D images of dental casts. (f) Computed tomography of the temporomandibular joint. (g) Magnetic resonance imaging of the articular discs of the temporomandibular joint.

**Figure:**
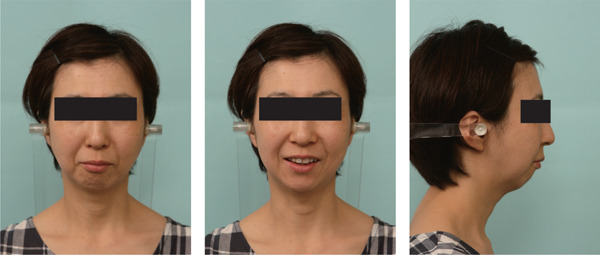
(a)

**Figure:**
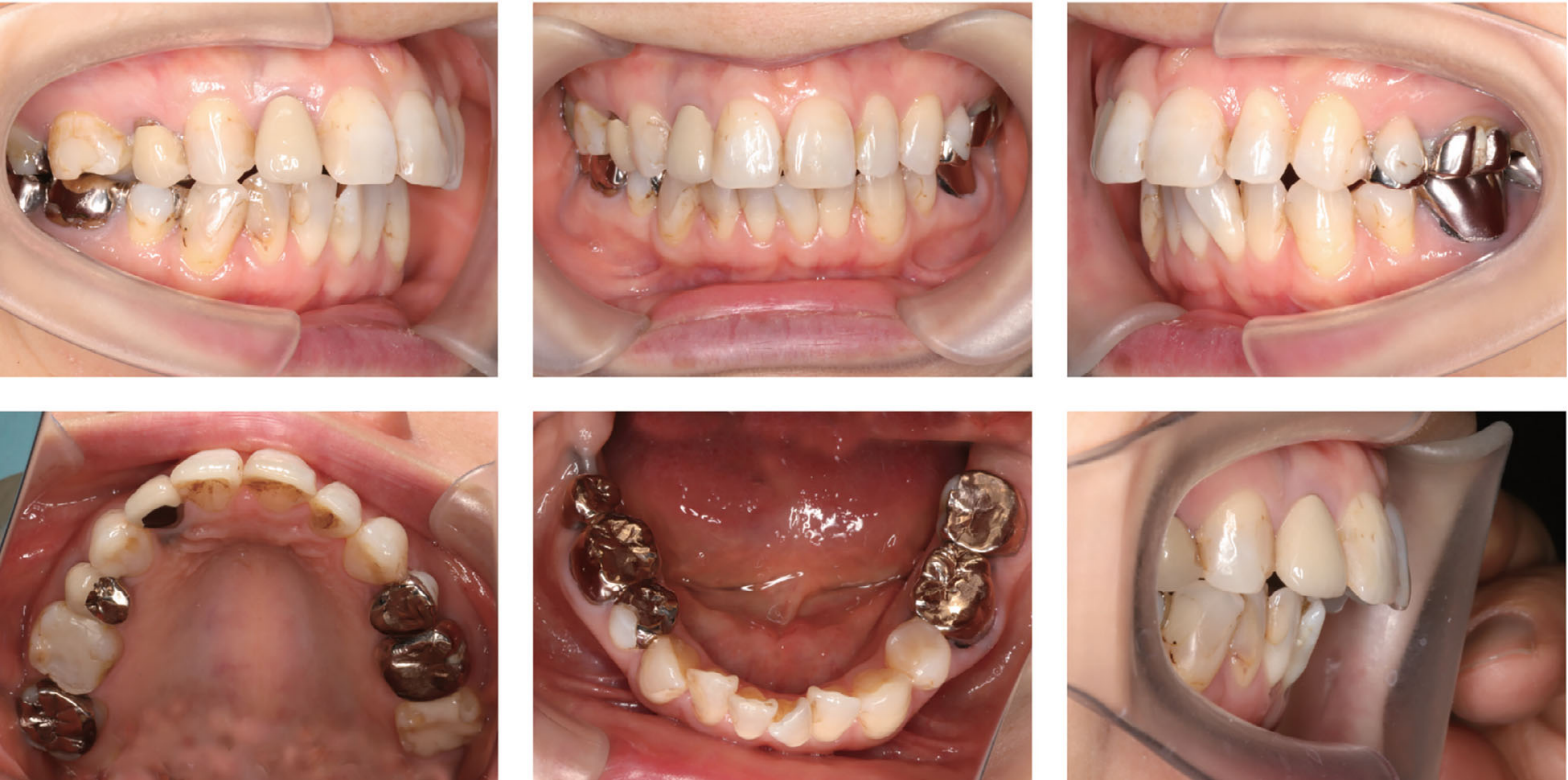
(b)

**Figure:**
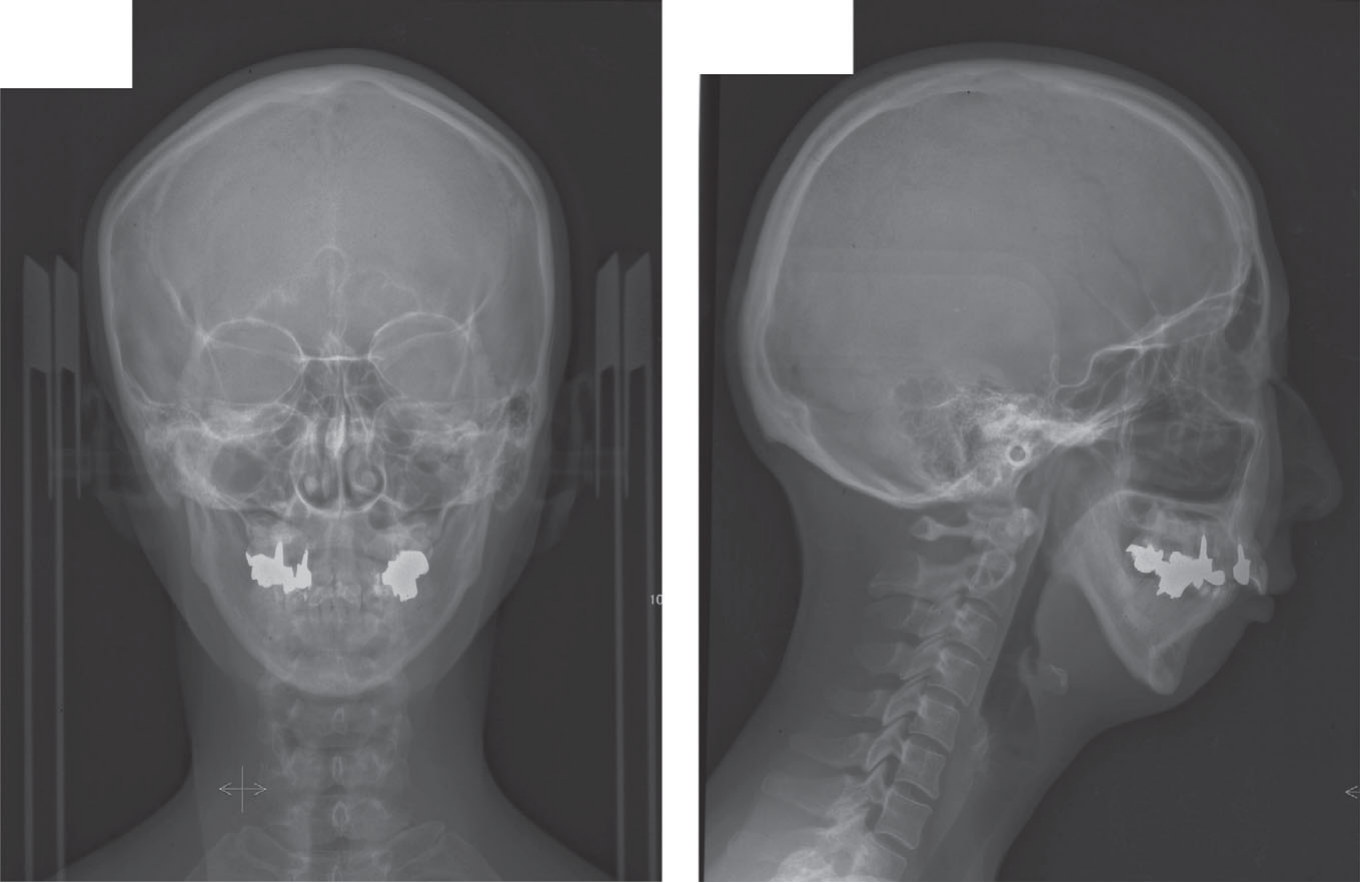
(c)

**Figure:**
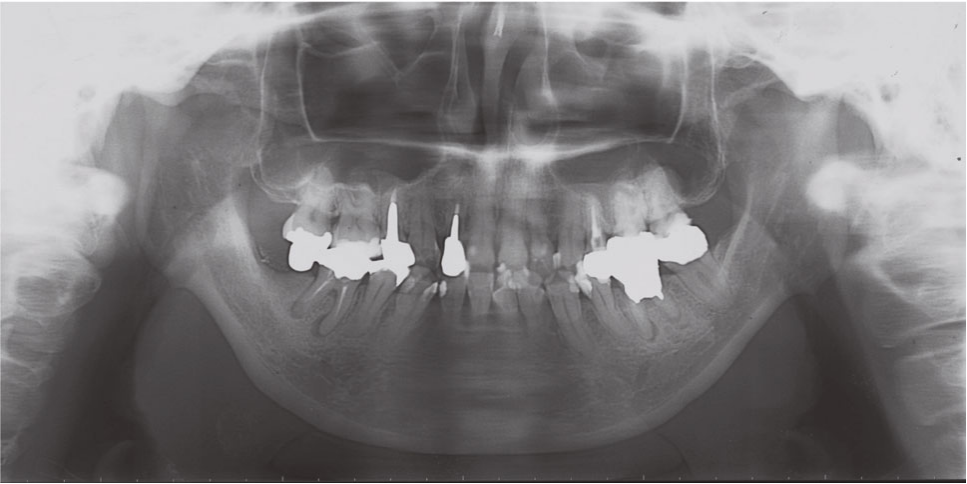
(d)

**Figure:**
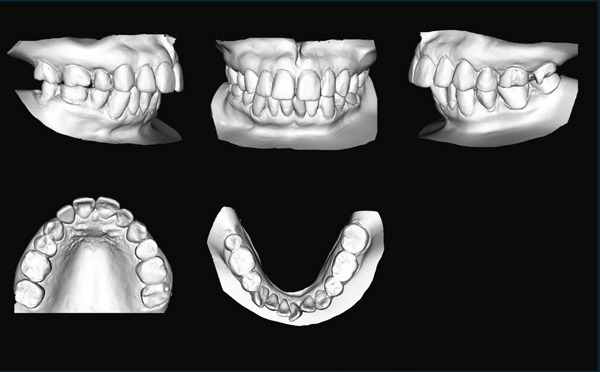
(e)

**Figure:**
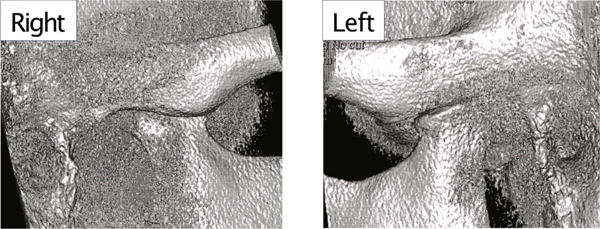
(f)

**Figure:**
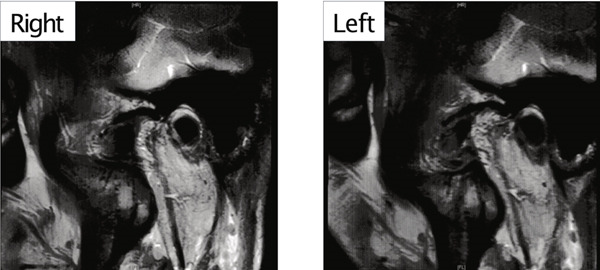
(g)

**Table 1:** Cephalometric measurements of pretreatment (T1), posttreatment (T2), and 21 months after treatment (T3).

**Measurements**	**Norm**	**T1**	**T2**	**T3**
Facial angle	84.8	73.4	80.5	80.0
Convexity	7.6	22.0	−0.8	−1.0
*A*–*B* plane	−4.5	−12.0	−4.8	−7.0
FMA	28.8	50.5	41.9	41.9
IMPA	96.3	87.1	88.6	86.6
SNA	82.3	76.0	71.5	71.6
SNB	78.9	67.5	69.5	68.9
ANB	3.4	8.5	2.0	2.7
U1 to FH	111.1	107.0	106.4	106.3
Gonial angle	122.2	127.0	127.0	127.0

Cone beam computed tomography (CBCT) images showed severe bilateral mandibular condylar head deformities (Figure 1). Magnetic resonance imaging (MRI) revealed severely deformed TMJ discs, displaced anteriorly without reduction on both sides (Figure 1). Due to the patient’s perception of nocturnal apnea, she was referred to the pulmonary hospital, where an overnight polysomnography test was performed, revealing an AHI of 43.3 events/h and minimum SpO_₂_ of 82.0% (Table 2). The patient was diagnosed with Angle Class II, skeletal Class II, mandibular retrognathia and hyperdivergent skeletal pattern with a steep mandibular plane, severe deformity of the bilateral condylar heads, and severe OSA. CPAP treatment was immediately initiated at the pulmonary hospital.

**Table 2:** Results of respiratory function test at pretreatment (T1) and posttreatment (T2).

**Measurements**	**T1**	**T2**
Apnea index	23.7	1.2
Hypopnea index	19.6	1.1
Apnea–hypopnea index (AHI)	43.3	2.3
Maximum apnea duration	77.8	64.8
Lowest saturation (%)	82.0	93.3

### 2.1. Treatment Objectives

The treatment objectives were to improve the intermaxillary relationship, establish a favorable anterior overjet and overbite, achieve an aesthetically acceptable profile, reduce TMJ stress to prevent the progression of condylar resorption, and improve respiratory function as much as possible.

### 2.2. Treatment Alternatives

Several alternative treatment plans were considered to accomplish the treatment goals. The first option focused solely on managing respiratory function with CPAP due to concerns about periodontal stress and possible progression of condylar head resorption. Particular attention was given to whether the periodontal tissues could withstand tooth movement. Orthodontic or orthognathic interventions on the mandibular bone carried a risk of aggravating condyle resorption and causing retrognathia recurrence.

The second approach was MMA surgery. This involves moving the maxilla anteriorly *via* Le Fort I osteotomy and advancing the mandible anteriorly using sagittal split ramus osteotomy (SSRO), aimed at enlarging the airway by repositioning both jaws. Since the patient′s maxilla was positioned posteriorly relative to the skull base, MMA seemed suitable. However, there was a concern regarding a substantial anterior shift of the mandible—over 5 mm—which might lead to recurring condyle resorption.

As a third option, surgeons recommended not moving the mandible anteriorly with SSRO. Instead, they proposed improving the intermaxillary relationship and profile by rotating the mandible counterclockwise, achieved by repositioning the maxilla upward and backward. This autorotation was anticipated to expand the airway, and combining the procedure with genioplasty could address mandibular retrognathia.

After consultation with the orthodontist and oral surgeon, the patient selected the third treatment option and provided written informed consent.

### 2.3. Treatment Progress

The maxillary and mandibular teeth were bonded with preadjusted edgewise brackets (0.018 × 0.025‐in. slot). Initial leveling of the upper and lower dentition was performed using the 0.016‐in. and 0.014‐in. round nickel–titanium wires, respectively. Interproximal reduction of the mandibular incisors was performed to regain space for alignment. Preoperative orthodontic treatment was performed for 19 months using a sequential nickel–titanium alloy wire and a 0.016 × 0.022‐in. stainless steel arch wire to align the maxillary and mandibular dentition before surgery.

**Figure 2:**
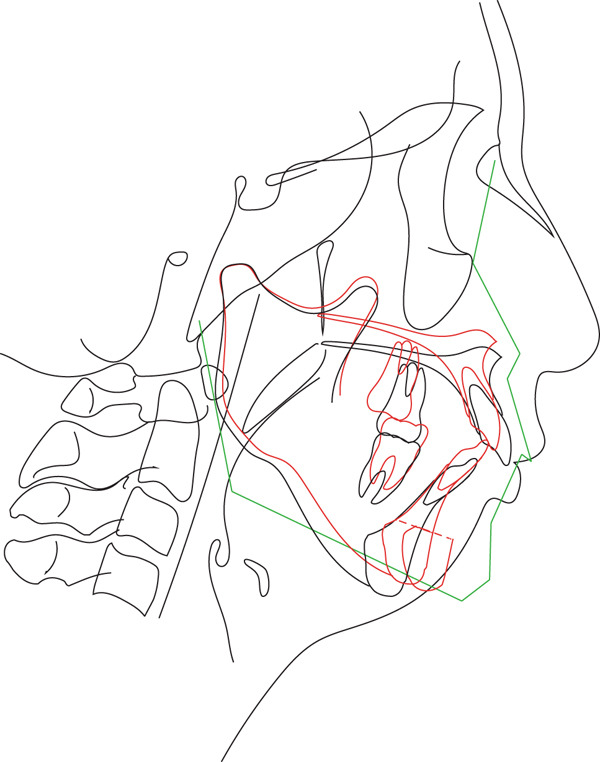
Cephalometric prediction of Le Fort I osteotomy and genioplasty.

After preoperative orthodontic treatment, Le Fort I osteotomy and genioplasty were performed under general anesthesia. Cephalometric predictions guided the procedures: the maxilla was moved 3.0 mm superiorly and 1.5 mm posteriorly for the anterior nasal spine and 4.0 mm superiorly and 1.0 mm posteriorly for the posterior nasal spine (PNS; Figure 2). The amount of mandibular autorotation (MA) was estimated to be 8.0° counterclockwise, and the chin was advanced by 6.0 mm during genioplasty.

During surgery, a Le Fort I osteotomy was performed to elevate the maxilla by cutting the bony structures forming the roof of the nasal cavity, followed by careful trimming of its floor. The surgeon directly inspected for any interference between the nasal septal cartilage and the nasal floor; when contact occurred, minor adjustments were made to prevent septal deviation. No deviation of the nasal septum or subsequent airway obstruction was noted.

The surgical design intentionally included the mental spine—attachment point for geniohyoid and genioglossus muscles—in the advanced bone fragment, preserving muscular attachments throughout the procedure. To stabilize the hyoid bone′s anterior–superior suspension, the geniohyoid and genioglossus muscles were sutured to the advanced bone before repositioning. Thus, the forward and upward movement of the hyoid bone was attributable to the traction generated by these muscles attached to the advanced segment.

Postoperative intermaxillary elastics were not used to reduce the stress on the TMJ. After 8 months of postoperative orthodontic treatment, all appriances were removed (Figure 3) and removable upper and lower retainers were applied. The patient’s respiratory function was re‐evaluated at the start of retention. A hook was placed to prevent extrusion of the right maxillary second molar.

Figure 3Posttreatment (T2) records. (a) Facial photographs. (b) Intraoral photographs. (c) Lateral and anteroposterior cephalograms. (d) Orthopantomograms. (e) 3D images of dental casts.

**Figure:**
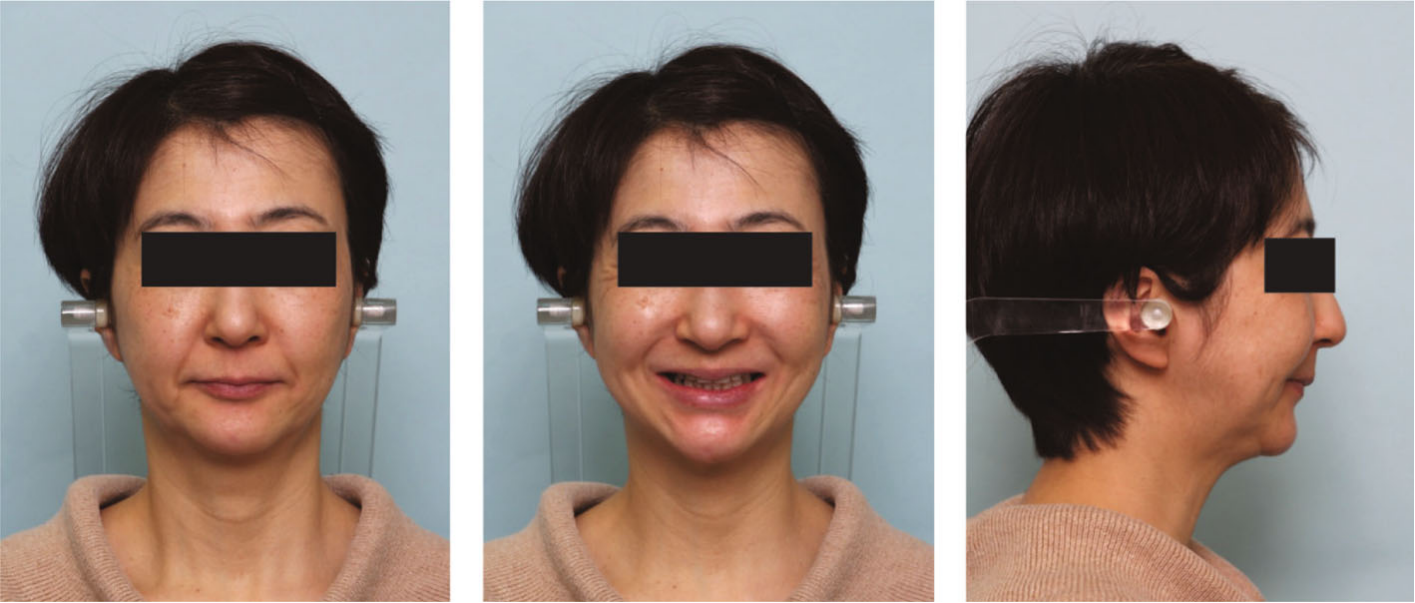
(a)

**Figure:**
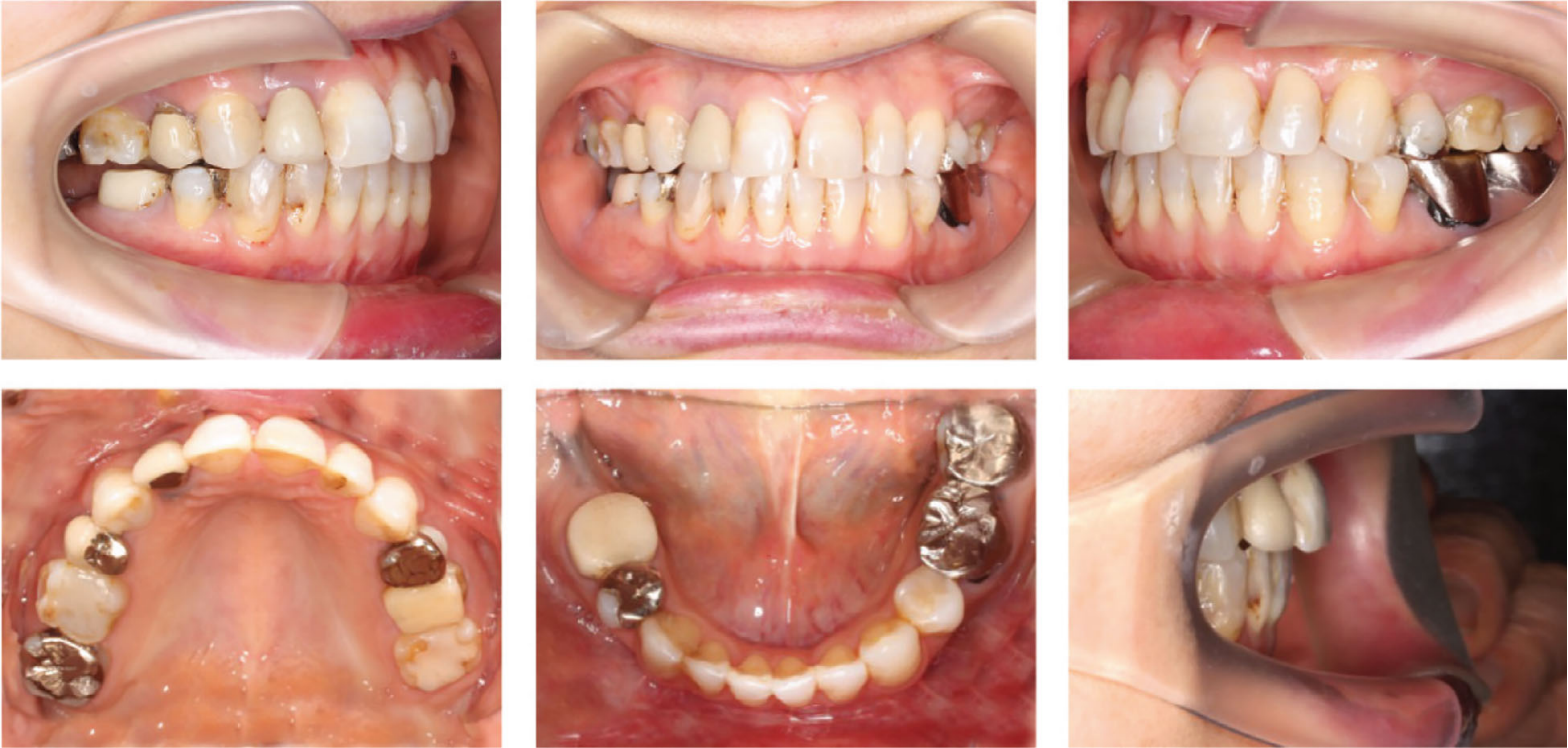
(b)

**Figure:**
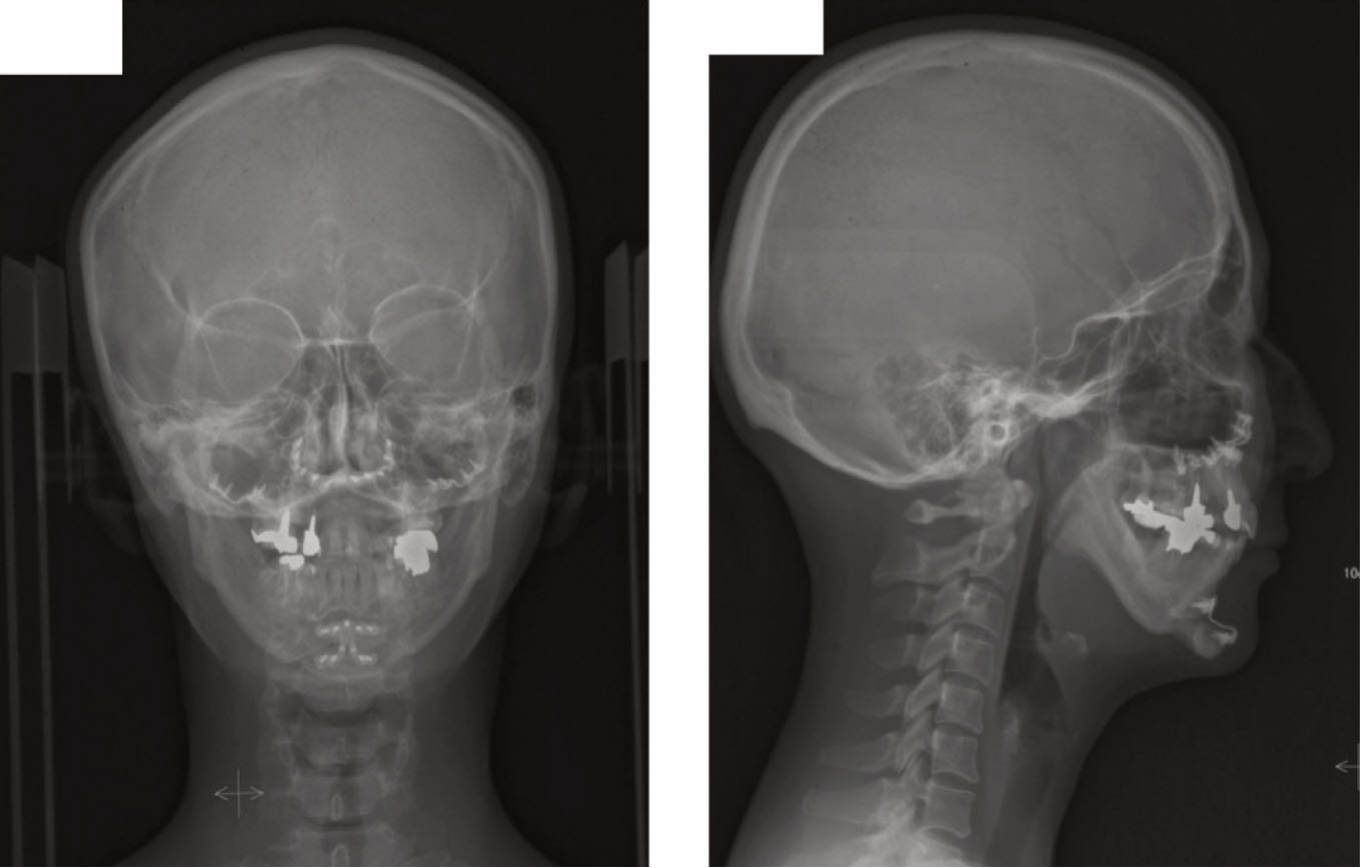
(c)

**Figure:**
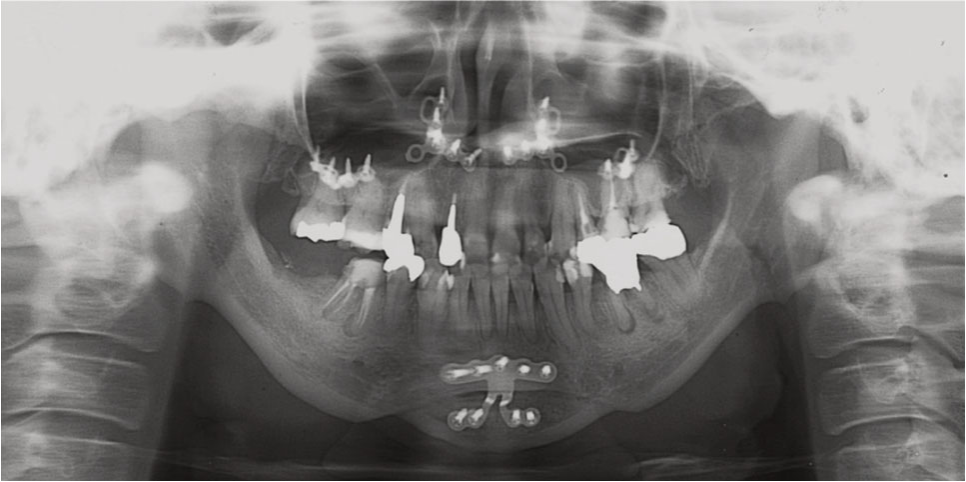
(d)

**Figure:**
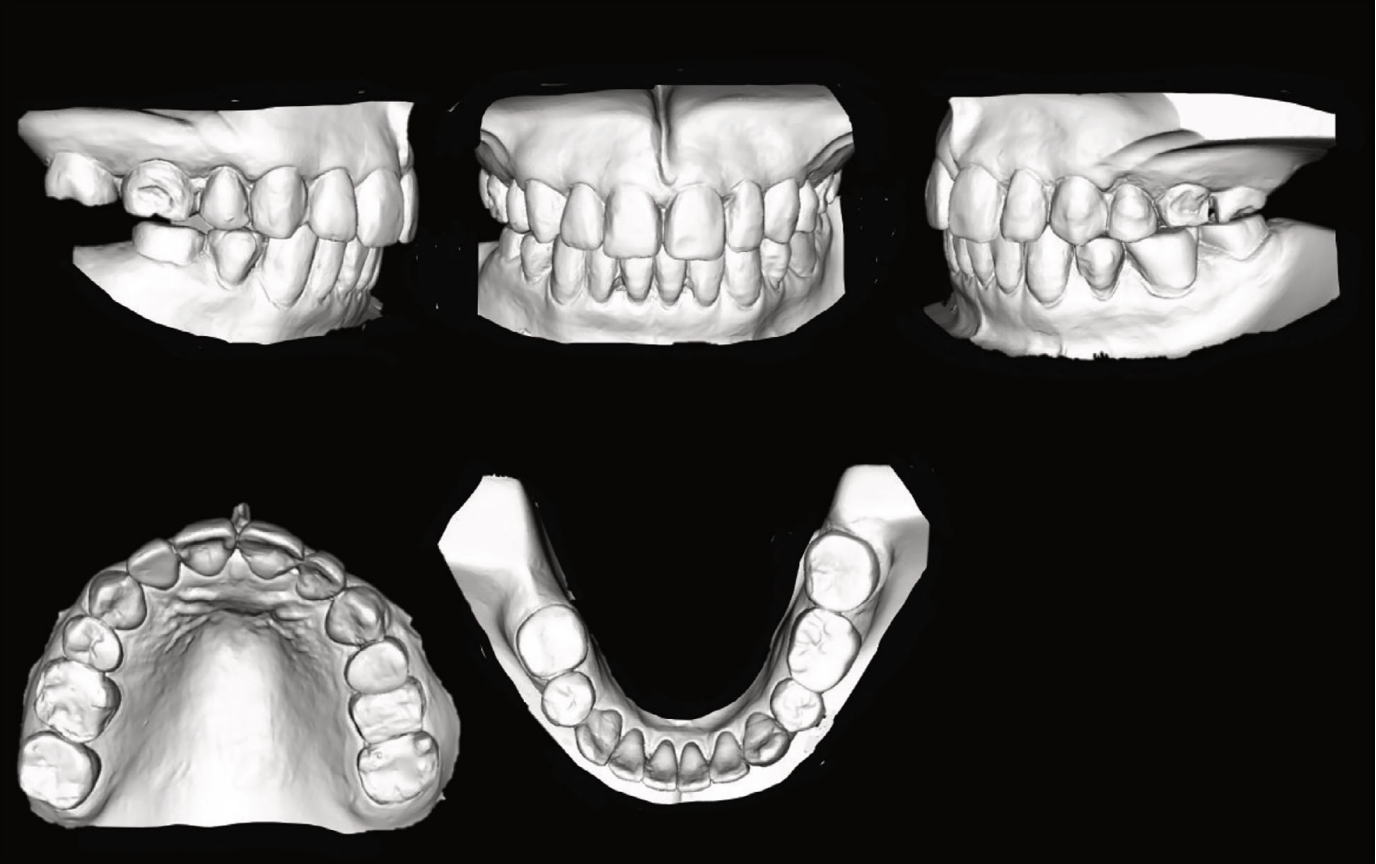
(e)

During a retention period of 21 months, the maxillary right second bicuspid and mandibular left first molars were extracted, and a bridge was placed (Figure 4).

Figure 421 months after treatment (T3) records. (a) Facial photographs. (b) Intraoral photographs. (c) Lateral and anteroposterior cephalograms. (d) Orthopantomograms. (e) 3D images of dental casts.

**Figure:**
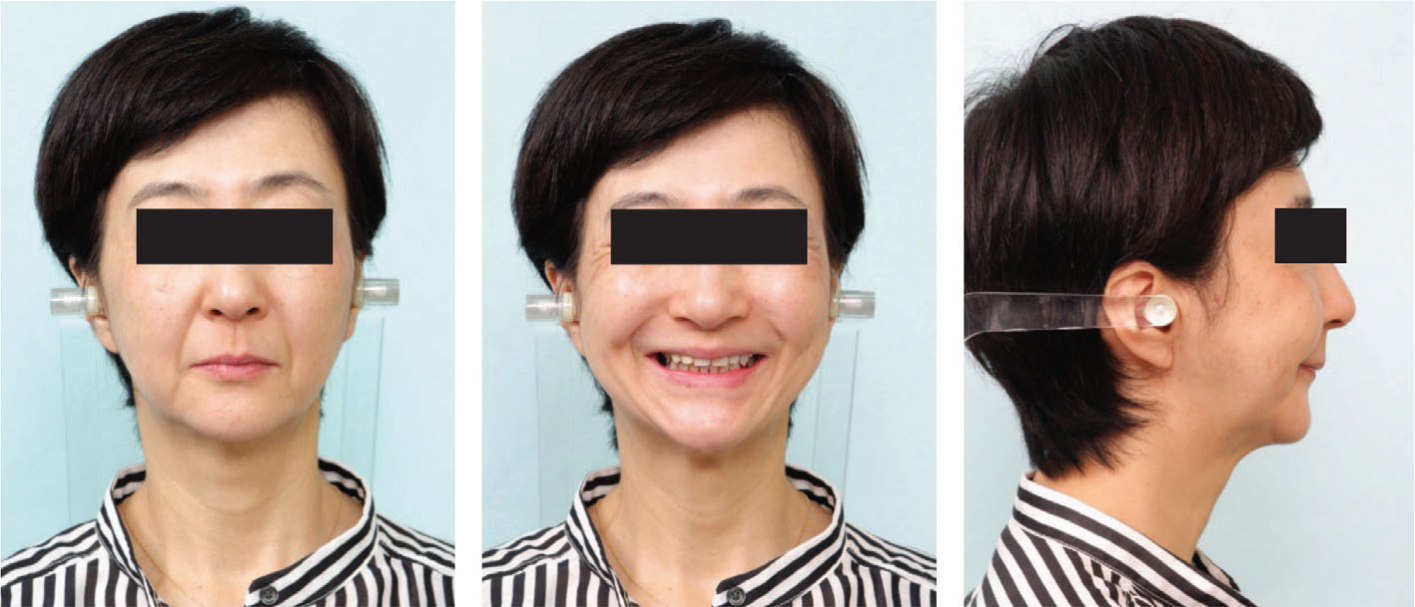
(a)

**Figure:**
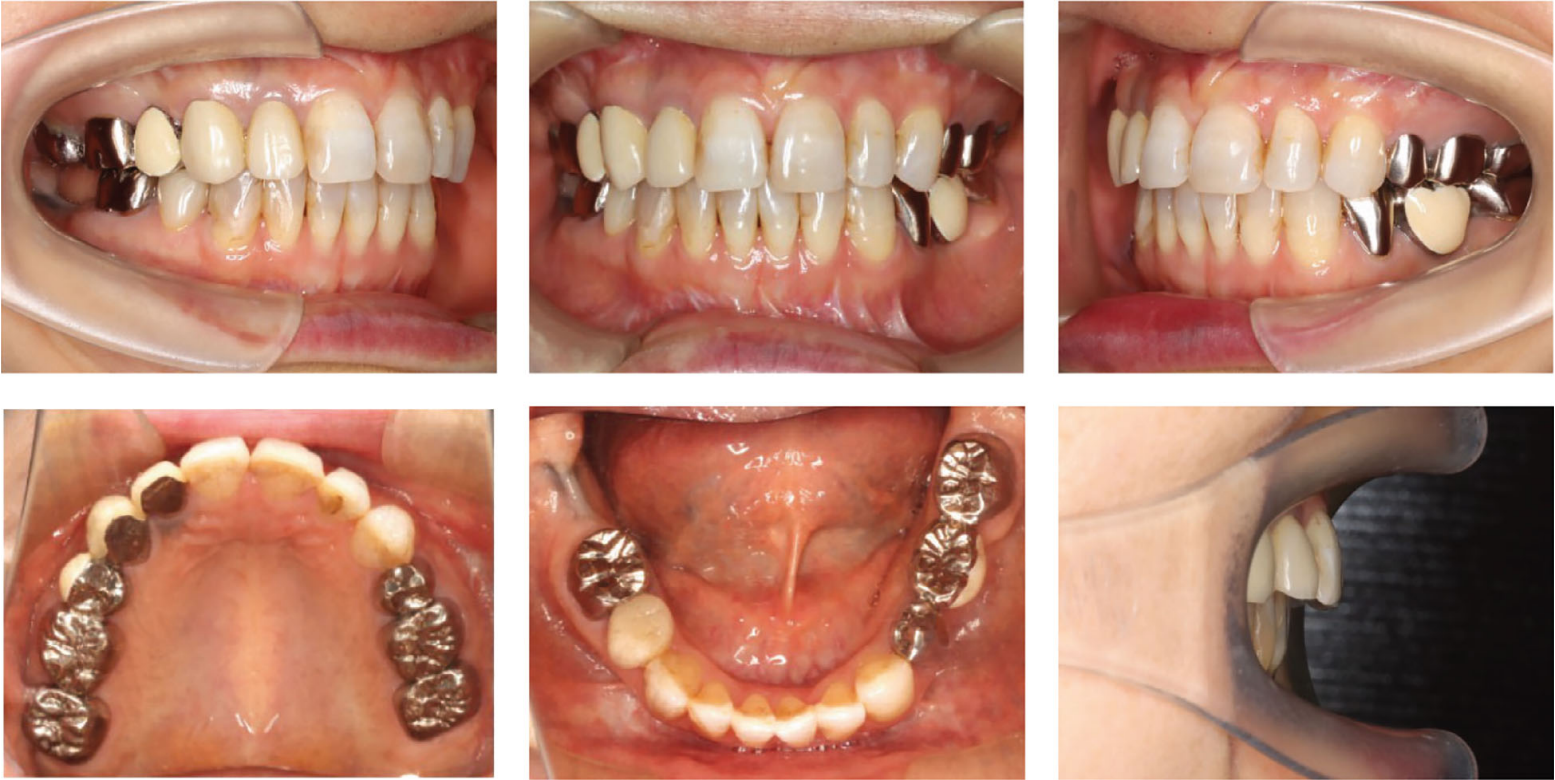
(b)

**Figure:**
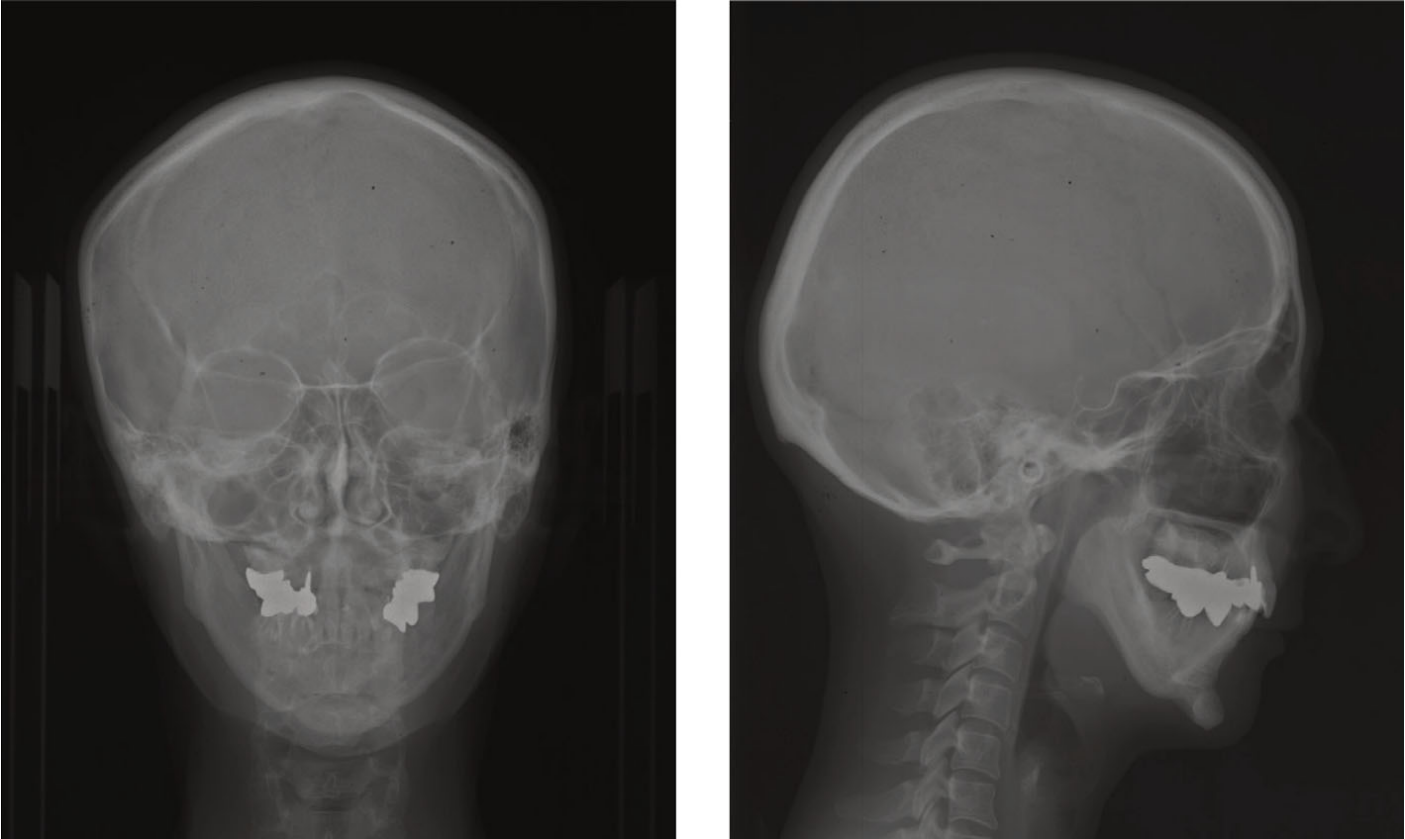
(c)

**Figure:**
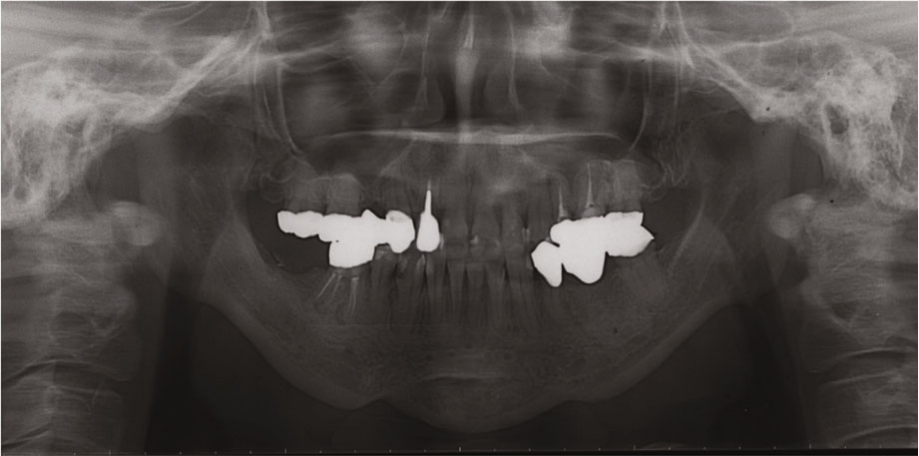
(d)

**Figure:**
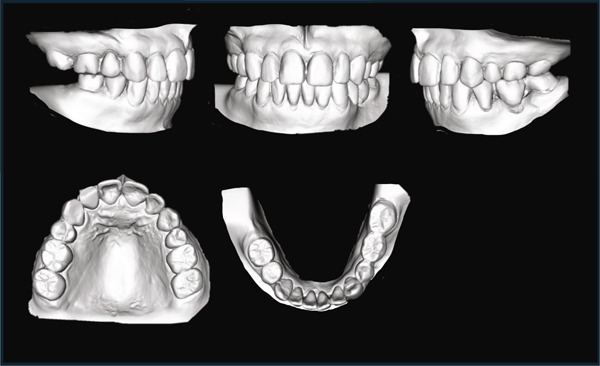
(e)

### 2.4. Treatment Results

The hypertonic mentalis muscular contraction during lip closure was resolved, and the lateral profile was improved (Figure 3). The anterior overjet and overbite were +2.0 mm each, and the molar relationship was Class I on the right and Class III on the left (Figure 3). Cephalometric analysis showed that the FMA was 41.9°, a decrease of 8.6° from the initial examination. The ANB had changed from +8.5° to +2.0°, indicating an improvement in the intermaxillary relationship (Table 1).

**Figure 5:**
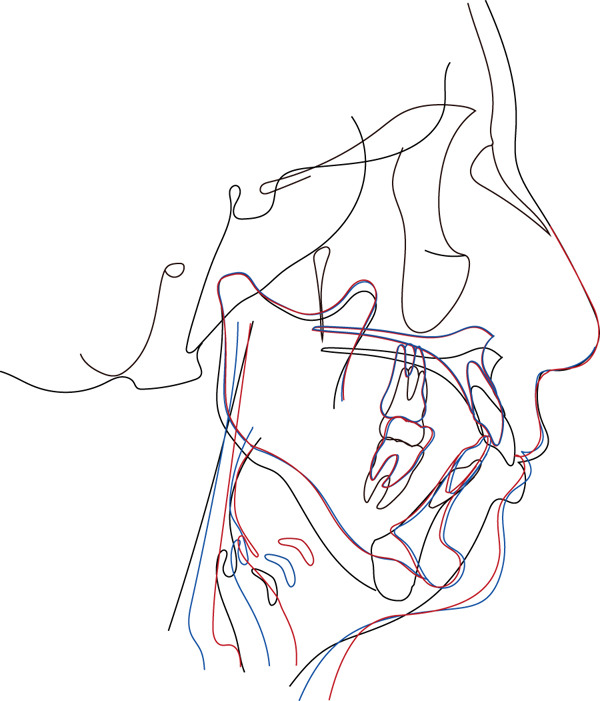
Cephalometric superimposition. Pretreatment (T1), black line; posttreatment (T2), red line; postretention (T3), blue line.

Superimposed lateral cephalograms showed the maxilla shifted posteriorly and upward from pretreatment (T1) to posttreatment (T2), reflecting the surgical plan (Figure 5). The mandible was rotated counterclockwise by 8.6°, and the chin was moved forward. The position of the hyoid bone changed to anterior and superior. From T2 to the postretention stage (T3), the mandibular condyle moved slightly posteriorly and upward to its pretreatment position, and the mandibular incisors slightly tilted lingually (Figure 5). In addition, the position of the hyoid bone continued to shift upward and anteriorly.

Drawing on a report by Ioi et al. on Japanese patients [19], we examined soft tissue changes in the maxillofacial region at T1, T2, and T3. Six parameters showed improvements following treatment: nasal prominence angle, nasolabial angle (Cm‐Sn‐Ls), upper and lower lip protrusions, facial convexity, and *Z* angle (Figure 6). These improvements suggest the patient acquired a well‐balanced maxillofacial soft tissue profile and improved upper and lower lip morphology posttreatment. Superimposed CT images taken immediately before and 1 year postorthognathic surgery revealed the chin advanced by over 10 mm (Figure 6).

Figure 6Analysis of soft tissue morphology. (a) Morphological change of profile soft tissue analysis. T1: green; T2: orange; and T3: yellow. (b) Superimposition of the computed tomography images of facial soft tissue just before orthognathic surgery (green) and 1 year after orthognathic surgery. Changes in airway area (c) and airway volume (d) calculated from the computed tomography images taken just before orthognathic surgery and 1 year postorthognathic surgery. After the three‐dimensional reconstruction from the CT images, the airway regions were segmented. The following planes were then defined and cut: the superior boundary (the plane parallel to the FH plane that passed through the PNS) and the middle and inferior boundaries (the soft palate and epiglottal planes, which were parallel to the FH plane and passed through the tip of the soft palate and the base of the epiglottis, respectively). (e) Superimposition of three‐dimensional images of the mandibular bones before surgery (green), postsurgery (blue), and 1 year pos‐surgery (orange).

**Figure:**
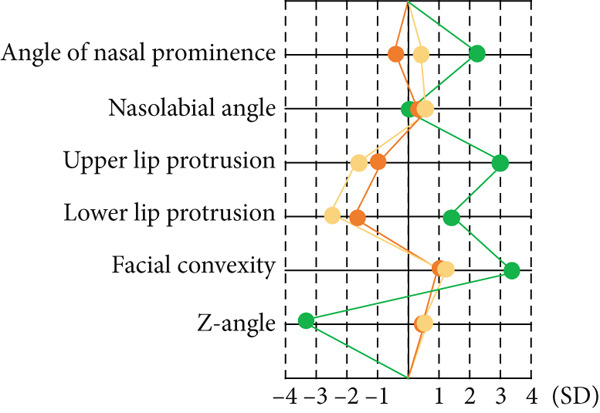
(a)

**Figure:**
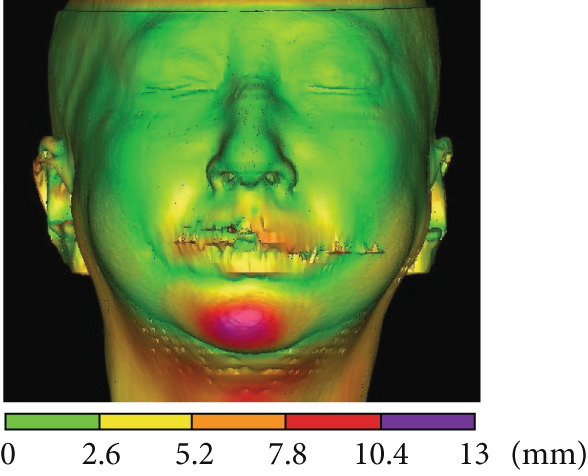
(b)

**Figure:**
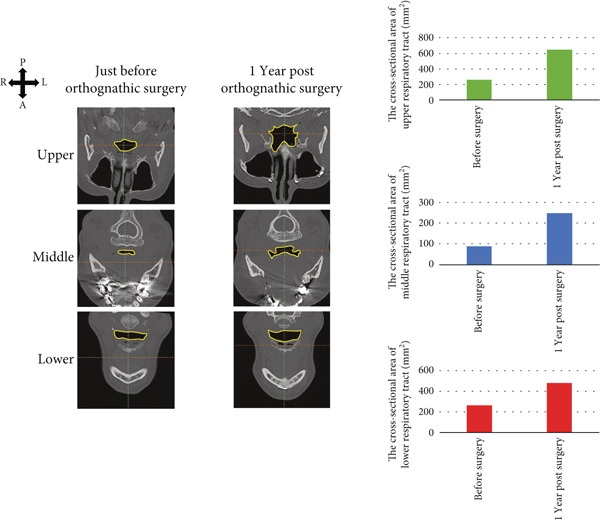
(c)

**Figure:**
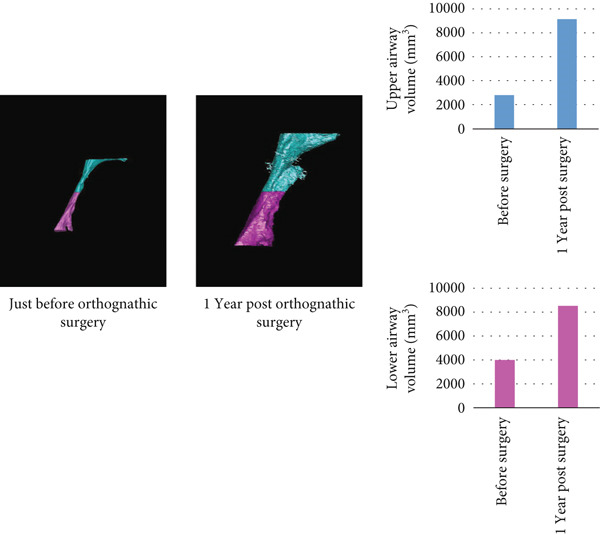
(d)

**Figure:**
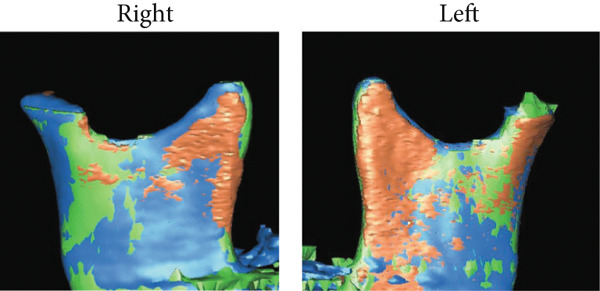
(e)

The cross‐sectional area of the upper respiratory tract was calculated from CT images taken immediately before and 1 year postsurgery as previously reported [20]. The cross‐sectional areas of the upper, middle, and lower respiratory tracts increased after surgery (Figure 6). Three‐dimensional analysis also revealed volume increases of 225.5% in the upper airway and 113.7% in the lower airway after surgery, consistent with earlier findings [21] (Figure 6). Moreover, the posttreatment (T2) respiratory function test found an AHI of 2.3, indicating an improvement from the pretreatment measurement (Table 2), allowing CPAP therapy to be discontinued. Finally, CT scans of the condyle showed no apparent morphological changes during treatment (Figure 6).

## 3. Discussion

We report a patient with retrognathia and hyperdivergence, presenting severe OSA and mandibular condylar deformity. Management included Le Fort I osteotomy and genioplasty, employing a multidisciplinary approach to reduce TMJ burden and achieve proper occlusion, intermaxillary relationships, facial profile, and respiratory function. The absence of obesity indicated that mandibular retrusion due to condylar resorption was likely the primary cause of OSA, as it narrowed the airway.

Recent studies have reported significant craniofacial morphological differences between individuals with and without OSA. Maxillary and mandibular size and positioning abnormalities contribute to increased OSA prevalence, with mandibular retrognathia having a particularly marked effect, especially in Asian populations [6, 7]. Patients with skeletal Class II have smaller upper airway volumes compared to Class I, while Class III tends to be associated with larger upper airway volumes compared with Classes I and II [22, 23]. Despite these observations, OSA remains in a multifactorial condition, necessitating comprehensive evaluation of upper airway volume in conjunction with other risk factors.

The relationship between upper airway anatomy and respiratory function is complex. Respiratory autoregulation suggests that structural assessment alone does not provide a complete evaluation of functional capacity. Others have reported that counterclockwise MA can enlarge superior nasal airway volume without improving airflow or resistance [24]. Similarly maxillary distraction osteogenesis *via* Le Fort I osteotomy significantly increases airway dimensions without enhancing the AHI [25]. Bimaxillary orthognathic surgery for Class IV patients has been associated with substantial pharyngeal airway narrowing, yet no increase in AHI—a key parameter for assessing OSA syndrome [26].

No apparent morphological changes were observed in the mandibular condylar head during active orthodontic treatment in the presented case. It was presumed that the patient developed idiopathic condylar resorption (ICR) or progressive condylar resorption (PCR) during adolescence, resulting in condylar deformity. ICR/PCR more commonly affects adolescent girls and is thought to be related to abnormal beta‐estradiol secretion [27]. These conditions result in progressive resorption of the mandibular condyle, considerably reducing the height of the mandibular ramus [28]. This reduction leads to clockwise rotation of the mandible, producing mandibular retrognathia with an anterior open bite and jaw muscle imbalance [29]. Patients with preoperative condylar deformity, hyperdivergent skeletal pattern, mandibular advancement of at least 5 mm, and short posterior mandibular height are at high risk of postsurgical condylar resorption [30]. Nevertheless, a report evaluating the long‐term stability of 15 patients who underwent Le Fort I osteotomy and mandibular rotation concluded that this surgical technique could minimize mandibular instability in patients prone to postoperative condylar resorption [31]. Meanwhile, Xiong et al. reported no association between mandibular counterclockwise rotation and condylar remodeling or skeletal relapse [32]. Successful treatment of skeletal and Angle Class II malocclusion, condylar deformity, gummy smile, and OSA has been achieved through orthognathic surgery involving maxillary upward movement and mandibular counterclockwise rotation and advancement [33].

A slight mandibular relapse was noted in the current case during the retention period (Figure 5), likely due to condylar resorption and displacement resulting from excessive MA. Superimposed pre‐ and postoperative 3D‐CT scans of the mandibular condyle revealed subtle morphological changes on the left side (Figure 6). However, the potential implications of even minor long‐term mandibular relapses after maxillary orthognathic surgery with MA must be carefully considered [32]. MA is also recognized as a strategy to reduce overload on the condylar head in class II malocclusion [34]. The rotational center of the mandible, located posterior and inferior to the condyle, can significantly affect outcomes dues to its variability [35]. Increased MA has been correlated with greater vertical displacement of the condylar joint [32], and excessive MA may exert a downward extension force on the condyle, potentially overstraining the attached musculature. This may be due to occlusal interferences involving the molars [34] and signifies possible risk for early postoperative relapses.

Joint effusion, indicating the accumulation of synovial fluid, blood, or other substances within the joint cavity, is commonly observed on MRI in patients TMJ disorders [36]. This suggests a relationship with the condition of the TMJ and related pain [37]. Research supports that joints with bone erosion in degenerative TMJ disorder have greater joint fluid volume compared to those with non‐repositionable disc displacement [38, 39].

In the current case, joint effusion was suspected based on T2‐weighted MRI scans at the initial visit (Figure 1g), with persistent findings throughout the retention period. Slight condylar resorption was also noted on the left side (Figure 6e). Ongoing monitoring is thus essential, not only for anterior–posterior relapses but also for lateral mandibular deviation. The articular discs exhibited signs of atrophy, deformation, with non‐reducible anterior displacement. Preoperative CT imaging revealed bone erosion in both mandibular condyles and osteophyte formation on the left side. However, no cases of trismus or TMJ pain were observed.

Although this is a single report, it highlights the importance of thoroughly analyzing respiratory function and the morphology and volume of craniofacial soft and hard when reconstructing occlusion in patients with retrognathia. However, the direction and magnitude of maxillary and mandibular movement through orthognathic surgery should be carefully considered for each patient. The effects of maxillofacial morphology and skeletal changes on respiratory function remain unclear, highlighting the need for further research to establish more effective and safer treatment strategies for patients with OSA.

## 4. Conclusions

In this report, we present a multidisciplinary approach for a hyperdivergent retrognathic patient with severe condylar deformity and OSA. Counterclockwise mandibular rotation combined with genioplasty can improve craniofacial morphology and respiratory function. Although OSA is a multifactorial disease, enlarging the upper airway may help improve symptoms. Nevertheless, as this is a single case report, further research is needed to clarify the direct relationship between craniofacial skeletal changes and respiration.

**Nomenclature**
ANB: A point‐nasion‐B point angle
FMA: Frankfort‐mandibular plane angle
IMPA: incisor mandibular plane angle
SNA: sella‐nasion‐A point angle
SNB: sella‐nasion‐B point angle
U1 to FH: upper central incisor to the Frankfort horizontal

## Ethics Statement

This case report obtained ethical approval from the Ethical Review Committee of the Institute of Science Tokyo (Approval Number D2014‐002). This case report was prepared following the CARE guidelines.

## Consent

The patient gave written informed consent for orthodontic treatment and orthognathic surgery and the use of her clinical data and images for research and publication purposes.

## Disclosure

All authors approved the submitted version of the manuscript and took responsibility for any part of the work.

## Conflicts of Interest

The authors declare no conflicts of interest.

## Author Contributions

**Y.K.**: conceptualization, data curation, formal analysis, funding acquisition, investigation, methodology, project administration, validation, visualization, and writing—original draft. **Y.Y.**: data curation, formal analysis, investigation, methodology, visualization, and writing—original draft. **N.T.**: investigation, methodology, resources, validation, writing—review and editing. **T.Y.**: conceptualization, project administration, resources, and writing–review and editing. **N.H.**: conceptualization, methodology, project administration, resources, software, and writing—review and editing. **K.M.:** conceptualization, funding acquisition, methodology, project administration, resources, software, supervision, and writing—review and editing.

## Funding

This work was supported by JSPS KAKENHI (Grant Number JP19H03857).

## Data Availability

The data that supports the findings of this study is available from the corresponding author, Y.K., upon reasonable request.
